# Geographic Accessibility of Retail Cannabis in Northern California and Prenatal Cannabis Use During the COVID-19 Pandemic

**DOI:** 10.1001/jamanetworkopen.2022.44086

**Published:** 2022-11-29

**Authors:** Kelly C. Young-Wolff, Natalie E. Slama, Alisa A. Padon, Lynn D. Silver, Aurash Soroosh, Stacey E. Alexeeff, Sara R. Adams, Monique B. Does, Cynthia I. Campbell, Deborah Ansley, Amy Conway, Nancy Goler, Lyndsay A. Avalos

**Affiliations:** 1 Division of Research, Kaiser Permanente Northern California, Oakland; 2 Department of Psychiatry and Behavioral Sciences, University of California, San Francisco; 3 Public Health Institute, Oakland, California; 4 Regional Offices, Kaiser Permanente Northern California, Oakland

## Abstract

**Importance:**

Prenatal cannabis use is associated with health risks for mothers and their children. Prior research suggests that rates of prenatal cannabis use in Northern California increased during the COVID-19 pandemic, but it is unknown whether increases varied with the local cannabis retail and policy environment.

**Objective:**

To test whether pandemic-related increases in prenatal cannabis use were greater among pregnant individuals with greater retail availability of cannabis around their homes or among those living in jurisdictions that allowed storefront retailers.

**Design, Setting, and Participants:**

A cross-sectional, population-based time series study used data from pregnancies in the Kaiser Permanente Northern California health care system screened for cannabis use before (January 1, 2019, to March 31, 2020) and during (April 1 to December 31, 2020) the early COVID-19 pandemic. Proximity to the nearest retailer and number of retailers within a 15-minute drive from one’s home and local cannabis storefront retailer policy (banned vs permitted) were calculated. Interrupted time series models were fit using multiplicative and additive Poisson regression, adjusting for age and race and ethnicity.

**Exposures:**

The COVID-19 pandemic.

**Main Outcomes and Measures:**

Prenatal cannabis use based on universal urine toxicology tests conducted during early pregnancy at entrance to prenatal care.

**Results:**

The sample (n = 99 127 pregnancies) included 26.2% Asian or Pacific Islander, 6.8% Black, 27.6% Hispanic, 34.4% non-Hispanic White, and 4.9% other, unknown, or multiracial individuals, with a mean (SD) age of 30.8 (5.3) years. Prenatal cannabis use before (6.8%) and during (8.2%) the pandemic was associated with closer proximity to a retailer, greater retailer density, and residing in a jurisdiction that permitted vs banned retailers. There was a greater absolute increase in cannabis use from before to during the pandemic among those within a 10-minute drive (<10 minutes: adjusted rate difference [aRD], 0.93 cases/100 patients; 95% CI, 0.56-1.29 cases/100 patients; ≥10 minutes: aRD, 0.40 cases/100 patients; 95% CI, 0.12-0.68 cases/100 patients; interaction *P* = .02). Otherwise, relative and absolute rates increased similarly across categories of cannabis retailer proximity/density and local policy (interaction *P* > .05).

**Conclusions and Relevance:**

Prenatal cannabis use was more common among individuals living in areas with greater retail availability of cannabis. Although relative rates increased similarly during the pandemic regardless of local cannabis retail and policy environment, there was a larger absolute increase associated with living closer to a storefront cannabis retailer. Continued monitoring of local cannabis policy, the retail environment, and prenatal cannabis use is needed.

## Key Points

**Question:**

Did rates of prenatal cannabis use during the COVID-19 pandemic vary with the local cannabis retail and policy environment?

**Findings:**

In this cross-sectional study, 99 127 pregnancies were screened for prenatal cannabis use via urine toxicology testing before (January 2019 to March 2020) and during (April 2020 to December 2020) the COVID-19 pandemic. Larger absolute increases in prenatal cannabis use were associated with living within vs more than a 10-minute drive of a cannabis retailer.

**Meaning:**

The findings of this study suggest that living near a cannabis retailer may increase the risk for increases in prenatal cannabis use during periods of heightened distress.

## Teaser

This cross-sectional study examines the use of cannabis by individuals who were pregnant before vs during the COVID-19 pandemic in association with geographic availability.

## Introduction

The COVID-19 pandemic has contributed to increased depression and psychological distress,^1,2,3,4^ and 1 in 10 US adults reported starting or increasing substance use during the pandemic.^5^ Individuals who are pregnant have been particularly affected, with unique concerns about COVID-19–related health risks, substantial changes to obstetric care, and elevated pandemic-related depression and anxiety.^6,7^ Prenatal depression and anxiety are associated with cannabis use during pregnancy,^8^ and some individuals who are pregnant report using cannabis to relieve these symptoms.^9^

In California, where cannabis retailers were deemed essential businesses during the pandemic and retail sales increased substantially,^10^ rates of prenatal cannabis use increased by 25% during the first 9 months of the pandemic compared with the 15 months prior.^11^ Although California legalized recreational adult-use cannabis sales in 2018, cities and counties may ban retail sale or limit retailer density, and local policies vary widely. Research has shown that greater cannabis retail availability (greater density around one’s home, shorter distance to the nearest retailer) during the first year of legal recreational sales in California was associated with higher odds of prenatal cannabis use.^12^ However, it is unknown whether prenatal cannabis use increased more during the pandemic among individuals with greater access to storefront cannabis retailers.

Understanding how the cannabis retail and policy environment relate to prenatal cannabis use during the pandemic is critically important, as cannabis use during pregnancy is associated with perinatal health risks (eg, low birth weight) and risks of adverse offspring outcomes in childhood, including psychoticlike experiences, externalizing, attention, thought, and social problems.^13,14,15,16,17,18^ Using data from a large health care system with universal screening for prenatal cannabis use in 2019 and 2020, we tested the hypothesis that local policies that permitted vs banned cannabis storefront retailers and greater retail availability would be associated with elevated rates of prenatal use before and during the pandemic (the second and third years of legal sales of adult-use cannabis in California) and greater pandemic-related increases in prenatal use.

## Methods

Kaiser Permanente Northern California (KPNC) is a large multispecialty health care system serving more than 4 million diverse members representative of Northern California’s insured population.^19^ All pregnancies screened from January 1, 2019, to December 31, 2020 (N = 100 005) were eligible for inclusion.^11^ Individuals who were pregnant but without a valid address in the KPNC 35-county catchment area within 90 days of urine toxicology testing were excluded (n = 878 [0.9%]). The prepandemic period included toxicology tests from January 1, 2019, to March 31, 2020, and the postpandemic period included tests from April 1 to December 31, 2020. During the study period, California regulations, which were subsequently rescinded, permitted delivery from licensed retailers to anywhere in the state.^20^ The KPNC institutional review board approved this study and waived informed consent. Study procedures meet Health Insurance Portability and Accountability Act requirements and the 42 CFR Part 2 regarding medical records. On enrollment in the health plan, all KPNC members are informed that their data may be used for research. This study followed the Strengthening the Reporting of Observational Studies in Epidemiology (STROBE) reporting guideline.

### Measures

#### Prenatal Cannabis Use

Prenatal cannabis use was based on urine toxicology tests universally conducted at entrance to prenatal care (approximately 8 weeks’ gestation), to which patients consent. Positive tests were confirmed with a laboratory test. Screening tests were performed on a Beckman Coulter AU680 chemistry analyzer using the Emit II Plus Cannabinoid Assay from Siemens with a cutoff of 45 ng/mL. Confirmatory testing for the presence of the cannabis metabolite, 11-nor-9-carboxy-delta 9-tetrahydrocannabinol, was performed by liquid chromatography–tandem mass spectrometry for all positive immunoassay results. The confirmation test methodology was by liquid chromatography–tandem mass spectrometry on a triple quadrupole system with a cutoff for positivity of 15 ng/mL.

#### Cannabis Storefront Retailer Proximity and Density

The California Department of Cannabis Control (DCC) database for 2019 and 2020^21^ provided cannabis storefront retailer addresses and license dates (proxy for operation dates). Addresses and license dates of microbusinesses, which operate 3 or more retail, cultivation, manufacturing, and distribution activities,^22^ permitted to conduct storefront retail sales in 2019 were collected from Weedmaps^23^ and dates of license activity and address were confirmed with DCC data; 2020 microbusiness data were collected directly from the DCC.

For each address of individuals who were pregnant, we computed the drive time to all retailers within a 60-minute driving radius using ArcGIS Pro, version 2.2.4 (Esri). We then calculated the proximity to the nearest cannabis retailer (<10- or ≥10-minute drive) and density of retailers within a 15-minute drive (0, 1-5, or ≥6 retailers). We included retailers in operation from the date of the patient’s last menstrual period through the date of the urine toxicology test.

#### Cannabis Storefront Retail Policy

Local laws for cannabis storefront retailers (medical and/or adult use) at the time of the prenatal urine toxicology test were extracted from the Fyllo Cannaregs commercial regulatory database, complemented by verification on jurisdictions’ websites and municipal codes, and outreach to city or county staff, when needed.^24^ Laws were based on the policy effective for each patient’s jurisdiction and categorized as either storefront retailers affirmatively allowed or were silent (meaning they did not explicitly allow or prohibit storefront retailers, in which case state allowance laws applied) or affirmatively prohibited.

### Sociodemographic Characteristics

Electronic health records provided data on patients’ age (<25, 25 to <35, ≥35 years), self-reported race and ethnicity (Asian or Pacific Islander, Black, Hispanic, non-Hispanic White, and other, unknown, or multiracial), and patient geocoded home addresses. Data on race and ethnicity were included because of known differences in the prevalence of prenatal cannabis use across racial and ethnic groups.

### Statistical Analysis

We used the monthly rates of prenatal cannabis use to conduct interrupted time series (ITS) analyses with Poisson regression models to compare rates of cannabis use before vs during the pandemic.^25^ The outcome was the monthly count of pregnancies positive for cannabis use and the offset was the log of the number of individuals who were pregnant that tested that month. We fit multiplicative and additive Poisson models for each retailer and policy variable and used interaction terms to test for differences. Interrupted time series models were also adjusted for age and race and ethnicity to account for any differences in distributions over time.^26^ We report the adjusted rate ratio (aRR) and 95% CI for multiplicative models and the adjusted rate differences (aRD) and 95% CI for additive models. Preliminary ITS models indicated that rates were stable before and during the pandemic, with no evidence of month-to-month trends, and ITS models were run without time trend variables.

To display results, we plotted monthly rates of prenatal cannabis use standardized to age in 2020, race and ethnicity, and category of exposure variable. We conducted all analyses in SAS, version 9.4 (SAS Institute Inc). Two-sided *P* values <.05 were considered statistically significant.

## Results

Race and ethnicity of the sample of 99 127 pregnancies (94 566 unique individuals) was 26.2% (n = 25 985 pregnancies) Asian or Pacific Islander, 6.8% Black (n = 6743 pregnancies), 27.6% Hispanic (n = 27 393 pregnancies), 34.4% non-Hispanic White (n = 34 108 pregnancies), and 4.9% other, unknown, or multiracial (n = 4898 pregnancies), with a mean (SD) age of 30.8 (5.3) years and mean (SD) gestational age at toxicology test of 58.3 (35.6) days. The sample included 62 322 pregnancies before (62.9%) and 36 805 pregnancies (37.1%) during the pandemic. There were negligible differences in age, race and ethnicity, and gestational age at urine toxicology testing between the 2 time periods.^11^ Our study included 339 adult-use storefront cannabis retailers, including 28 microbusinesses (14 adult-use only, 29 medical-use only, 296 both adult and medical use).

### Overall

As previously reported, the standardized rate of prenatal cannabis use was higher during (8.15%) vs before (6.75%) the pandemic (*P* < .001).^11^ The overall increase was statistically significant in both relative and additive Poisson models (Table).

**Table.** Rates of Prenatal Cannabis Use per 100 Pregnancies Before and During the COVID-19 Pandemic by the Cannabis Retail Environment and Local Policy^a^

Cannabis retail environment and local policy	Pregnancies, No. (%)		Standardized prenatal cannabis rate		Multiplicative model^b^		Additive model^b^	
	Before COVID-19	During COVID-19	Before COVID-19	During COVID-19	During COVID-19 vs before COVID-19, rate ratio (95% CI)	*P* value relative to reference	During COVID-19 vs before COVID-19, rate difference (95% CI)	*P* value relative to reference
Overall	62 322 (62.9)	36 805 (37.1)	6.75	8.15	1.20 (1.15-1.26)^c^	NA	0.67 (0.44-0.91)^c^	NA
Proximity: Drive time to nearest storefront retailer, min								
≥10	30 449 (48.9)	17 321 (47.1)	5.91	7.04	1.19 (1.10-1.28)^c^	Reference	0.40 (0.12-0.68)^c^	Reference
<10	31 873 (51.1)	19 484 (52.9)	7.55	9.13	1.21 (1.14-1.28)^c^	.72	0.93 (0.56-1.29)^c^	.02
Density: No. of retailers within ≤15-min drive								
0	20 218 (32.4)	10 810 (29.4)	5.85	6.84	1.17 (1.06-1.28)^c^	Reference	0.42 (0.08-0.75)^c^	Reference
1-5	17 231 (27.6)	10 155 (27.6)	7.08	8.72	1.23 (1.12-1.34)^c^	.44	0.98 (0.49-1.47)^c^	.06
≥6	24 873 (40.0)	15 840 (43.0)	7.25	8.67	1.19 (1.11-1.28)^c^	.68	0.70 (0.32-1.08)^c^	.26
Local cannabis storefront retail policy								
No storefront retail	29 900 (48.0)	17 075 (46.4)	6.20	7.53	1.21 (1.13-1.30)^c^	Reference	0.50 (0.18-0.83)^c^	Reference
Storefront retail	32 422 (52.0)	19 730 (53.6)	7.25	8.68	1.20 (1.12-1.27)^c^	.84	0.81 (0.47-1.14)^c^	.19

Abbreviation: NA, not applicable.

^a^ Rates are standardized to age in year 2020, race and ethnicity, and cannabis retail policy and environment status of the pregnancies in the overall study sample. Prenatal cannabis use was based on a positive toxicology screening conducted as part of standard prenatal care (at approximately 8 weeks’ gestation). Screening tests were performed on a Beckman Coulter AU680 chemistry analyzer using the Emit II Plus Cannabinoid Assay from Siemens with a cutoff of 45 ng/mL. Confirmatory testing for the presence of the cannabis metabolite, 11-nor-9-carboxy-delta 9-tetrahydrocannabinol, was performed by liquid chromatography–tandem mass spectrometry for all positive immunoassay results. The confirmation test methodology was liquid chromatography–tandem mass spectrometry on a triple quadrupole system with a cutoff for positivity of 15 ng/mL.

^b^ Poisson models were adjusted for age and race and ethnicity.

^c^ Significant at 2-sided *P* < .05.

### Retailer Proximity

Approximately half of pregnant individuals lived within a 10-minute drive of at least 1 cannabis retailer before (51.1%) and during (52.9%) the pandemic. Before the pandemic, rates of prenatal cannabis use were higher among those within a 10-minute (7.55%) vs 10-minute or more (5.91%) drive from the nearest retailer (*P* < .001) (Table; Figure, A). During the pandemic, rates were also greater among individuals within a 10-minute (9.13%) vs 10-minute or more (7.04%) drive from the nearest retailer (*P* < .001).

**Figure.**
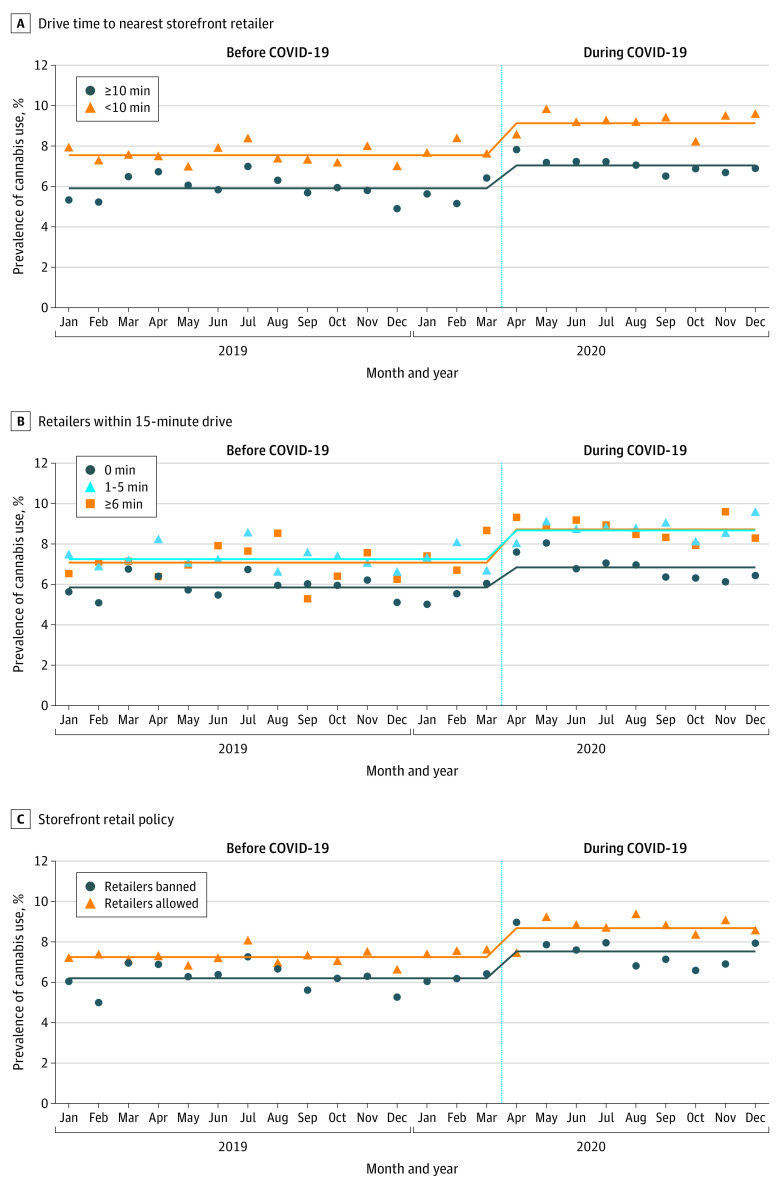
Rates of Prenatal Cannabis Use Before and During the COVID-19 Pandemic by the Cannabis Retail Environment and Local Policy Rates shown for drive time to nearest storefront retailer (A), retailers within 15-minute drive (B), and storefront retail policy (C). Prenatal cannabis use was based on a positive toxicology screening conducted as part of standard prenatal care (at approximately 8 weeks’ gestation). Screening tests were performed on a Beckman Coulter AU680 chemistry analyzer using the Emit II Plus Cannabinoid Assay from Siemens with a cutoff of 45 ng/mL. Confirmatory testing for the presence of the cannabis metabolite, 11-nor-9-carboxy-delta 9-tetrahydrocannabinol, was performed by liquid chromatography–tandem mass spectrometry for all positive immunoassay results. The confirmation test methodology was liquid chromatography–tandem mass spectrometry on a triple quadrupole system with a cutoff for positivity of 15 ng/mL.

Relative rates of prenatal cannabis use increased similarly from before to during the pandemic for individuals within a 10-minute drive (aRR, 1.21; 95% CI, 1.14-1.28) and 10-minute or more drive (aRR: 1.19; 95% CI, 1.10-1.28) to the nearest retailer (interaction *P* = .72). However, there was a greater absolute increase in cannabis use among those within a 10-minute drive (<10 minutes: aRD, 0.93 cases/100 patients; 95% CI, 0.56-1.29 cases/100 patients; ≥10 minutes: aRD, 0.40 cases/100 patients; 95% CI, 0.12-0.68 cases/100 patients; interaction *P* = .02).

### Retailer Density

The number of retailers within a 15-minute drive of individuals’ homes was similar before (0: 32.4%; 1-5: 27.6%; ≥6: 40.0%) and during (0: 29.4%; 1-5: 27.6%; ≥6: 43.0%) the pandemic. Before the pandemic, a higher percentage of pregnant individuals had a retailer within a 15-minute drive of their homes in jurisdictions that permitted (88.2%) vs banned (45.2%) storefront cannabis retailers. Similarly, during the pandemic, a higher percentage of individuals who were pregnant had a retailer within a 15-minute drive of their homes in jurisdictions that permitted (87.3%) vs banned (51.4%) storefront cannabis retailers. Rates of prenatal cannabis use were higher among those with a greater number of retailers before (0: 5.85%; 1-5: 7.08%; ≥6: 7.25%) and during (0: 6.84%; 1-5: 8.72%; ≥6: 8.67%) the pandemic (Table; Figure, B). Rates of prenatal cannabis use increased similarly from before to during the pandemic for all categories of retailer density (multiplicative models: aRR from 1.17 to 1.23, interaction *P* = .74; additive models: aRD from 0.42 to 0.98, interaction *P* = .89).

### Local Retail Policy

Before the pandemic, 34.0% of jurisdictions allowed storefront cannabis retailers and 52.0% of the sample lived in jurisdictions allowing storefront retailers. During the pandemic, 37.0% of jurisdictions allowed storefront retailers and 53.6% of the sample lived in jurisdictions allowing storefront retailers. Rates of prenatal cannabis use were higher among individuals in jurisdictions allowing vs banning these retailers before (7.25% vs 6.20%) and during (8.86% vs 7.53%) the pandemic (Table; Figure, C). Rates increased similarly from before to during the pandemic regardless of whether storefront retailers were allowed (multiplicative model: aRR, 1.20; 95% CI, 1.12-1.27; additive model: 0.81; 95% CI, 0.47-1.14) or banned (multiplicative model: aRR, 1.21; 95% CI, 1.13-1.30; additive model: 0.50; 95% CI, 0.18-0.83) (multiplicative model: interaction *P* = .84; additive model: interaction *P* = .19).

## Discussion

Rates of prenatal cannabis use in Northern California were higher before and during the pandemic among individuals with greater retail availability of cannabis and local policies permitting storefront retail sales. Long-standing tobacco and alcohol research has noted that greater retailer density is related to higher risk of substance use, and limits on retailers can reduce these risks.^27,28,29,30,31,32,33,34,35^ Greater exposure and easier access to storefront cannabis retailers may have contributed to an increased risk of prenatal cannabis use; however, retailers may also have opened more often in neighborhoods where cannabis use was more prevalent. Studies are needed to better understand how the cannabis retail environment affects prenatal use and to assess policies limiting retailers (eg, retailer caps, mandated distance between retailers).

Notably, rates of prenatal cannabis use increased similarly on the multiplicative scale for individuals who were pregnant during the pandemic regardless of the cannabis retail and policy environment. However, more relevant to public health,^36^ there were larger absolute increases in use during the pandemic among those living within vs more than a 10-minute drive of a retailer. Living in closer proximity to a cannabis retailer is potentially an important risk factor for greater increases in cannabis use among individuals who are pregnant during periods of heightened distress.

The lack of differences in increased relative rates of prenatal cannabis use during the pandemic associated with the local cannabis policy and retail environment may be because cannabis delivery was allowed anywhere in California during the study period (January 16, 2019, to November 18, 2020) under state regulation, before delivery ordinances returned to local control.^20^ Our study includes the early pandemic period, when individuals who were pregnant may have been especially likely to have cannabis delivered, regardless of how close a storefront retailer was to their homes. Results also highlight ubiquitous access to retailers during the second and third years of legal adult-use sales in California, regardless of local policies banning storefront retailers. Notably, during the early period of the pandemic, more than half (51.4%) of individuals living in a jurisdiction that banned cannabis retailers had 1 or more storefront retailer within a 15-minute drive from their homes. These factors may have made it more difficult to detect a true effect of the cannabis retail environment and local policy.

### Limitations

This study has several limitations. Results are limited to individuals who were pregnant receiving prenatal care in KPNC and may not generalize to uninsured individuals who were pregnant or to those outside of California. Prenatal cannabis use was assessed at entrance to prenatal care and does not reflect continued use throughout pregnancy. Furthermore, urine toxicology tests do not capture quantity, frequency, or cannabis product used. Cannabis is detectable in urine for approximately 30 days after the last use among individuals with heavy use. It is possible that some individuals with prenatal cannabis use only used before recognition of pregnancy. Finally, retail availability was assessed using storefront retailers only, many of whom also deliver, and we did not assess delivery-only retailers, as license data did not specify their served geographic area.

## Conclusions

The findings of this study suggest that cannabis retail proximity and density and local cannabis policies were important factors in the rates of prenatal cannabis use before and during the early COVID-19 pandemic. Rates of prenatal cannabis use from before to during the pandemic increased similarly on the multiplicative scale, regardless of the local retail and policy environment. However, there were larger absolute increases in prenatal cannabis use among individuals who were pregnant who lived closer to a storefront cannabis retailer. Continued monitoring of the role of local cannabis policies and the storefront and delivery retail environment is needed to better understand their association with cannabis use among individuals who are pregnant and other vulnerable populations.
